# Stress and Sucrose Intake Modulate Neuronal Activity in the Anterior Hypothalamic Area in Rats

**DOI:** 10.1371/journal.pone.0156563

**Published:** 2016-05-31

**Authors:** Arojit Mitra, Geneviève Guèvremont, Elena Timofeeva

**Affiliations:** Département de Psychiatrie et de Neurosciences, Faculté de Médecine, Centre de recherche de l’Institut universitaire de cardiologie et de pneumologie de Québec, Université Laval, Québec (QC), G1V 0A6, Canada; Technion—Israel Institute of Technology, ISRAEL

## Abstract

The anterior hypothalamic area (AHA) is an important integrative relay structure for a variety of autonomic, endocrine, and behavioral responses including feeding behavior and response to stress. However, changes in the activity of the AHA neurons during stress and feeding in freely moving rats are not clear. The present study investigated the firing rate and burst activity of neurons in the central nucleus of the AHA (cAHA) during sucrose intake in non-stressful conditions and after acute stress in freely behaving rats. Rats were implanted with micro-electrodes into the cAHA, and extracellular multi-unit activity was recorded during 1-h access to 10% sucrose in non-stressful conditions or after acute foot shock stress. Acute stress significantly reduced sucrose intake, total sucrose lick number, and lick frequency in licking clusters, and increased inter-lick intervals. At the cluster start (CS) of sucrose licking, the cAHA neurons increased (CS-excited, 20% of the recorded neurons), decreased (CS-inhibited, 42% of the neurons) or did not change (CS-nonresponsive, 38% of the neurons) their firing rate. Stress resulted in a significant increase in the firing rate of the CS-inhibited neurons by decreasing inter-spike intervals within the burst firing of these neurons. This increase in the stress-induced firing rate of the CS-inhibited neurons was accompanied by a disruption of the correlation between the firing rate of CS-inhibited and CS-nonresponsive neurons that was observed in non-stressful conditions. Stress did not affect the firing rate of the CS-excited and CS-nonresponsive neurons. However, stress changed the pattern of burst firing of the CS-excited and CS-nonresponsive neurons by decreasing and increasing the burst number in the CS-excited and CS-nonresponsive neurons, respectively. These results suggest that the cAHA neurons integrate the signals related to stress and intake of palatable food and play a role in the stress- and eating-related circuitry.

## Introduction

The anterior hypothalamic area (AHA) is located in the anterior medial zone of the hypothalamus. Based on the connections of this area and its functional implications, the AHA is thought to be an important integrative and relay structure for a variety of autonomic, endocrine, and behavioral responses [1,2]. The AHA receives dense innervation from the lateral septum (LS), bed nucleus of the stria terminalis (BNST), suprachiasmatic nucleus, subiculum and the ventromedial hypothalamic nucleus (VMH) [3]. A large ascending pathway from the AHA terminates in the LS [1,3–5], whereas a dense ventral pathway descends to the paraventricular (PVN) and periventricular hypothalamic nuclei [6], perifornical lateral hypothalamic area (LHA), and ventromedial and posterior hypothalamus, and reaches the periaqueductal gray and reticular formation [1]. It has been suggested that the AHA integrates information from the VMH and LS before relaying it to the periaqueductal gray [7]; moreover, the integrity of this pathway is important for full expression of defensive and flight behaviors [7–9]. Sympathetic activation and pressor response induced by immobilization stress were also found to be dependent on the activity of the AHA [10]. Moreover, the AHA mediates anxious behavior induced by activation of the LS neurons [2]. Injection of an anterograde transsynaptic tracer into the LS revealed the presence of tracer-labeled neurons in the AHA and the parvocellular PVN, while direct optogenetic inhibition of the LS projections to the AHA decreased stress-induced corticosterone plasma levels [2]. These results suggest that the AHA can modulate the activity of the hypothalamic-pituitary-adrenal (HPA) axis via its effects on the PVN hypophysiotropic neurons.

Along with the medial preoptic area and dorsomedial hypothalamic nucleus, the AHA contains the most prominent group of γ-aminobutyric acid (GABA) neurons in the hypothalamus [11]. The small- and medium-sized GABAergic neurons of the AHA are particularly dense in the central nucleus of the AHA (cAHA) [11], and a fraction of the GABAergic neurons in this area induces expression of c-Fos, a molecular marker of neuronal activation, in response to stress [12]. GABA neurotransmission is involved in food intake regulation [13–17]. Direct administration of the GABA_A_ receptor agonist muscimol into the AHA was found to increase feeding in satiated sheep [18]. In addition to GABA, the AHA neurons express a number of neurotransmitters involved in food intake regulation, such as melanin-concentrating hormone [19], somatostatin [20], enkephalin [21], dynorphin [22], galanin [23], substance P [24], oxytocin, and vasopressin [25]. Direct administration of α-melanocyte-stimulating hormone into the AHA significantly decreased food intake, while injection of agouti-related peptide significantly increased food intake in rats [26]. There is evidence that the AHA neurons are sensitive to sucrose intake. For example, scheduled access to sucrose induced the expression of c-*fos* mRNA in the AHA and adjacent anterior lateral hypothalamic area within a few hours preceding sucrose access, whereas consumption of sucrose decreased c-*fos* expression in these areas [27]. Following chronic stress, the AHA showed enhanced glutamic acid decarboxylase (GAD) 65 expression, which decreased on consumption of a sucrose solution [28]. Corticosterone resulted in a considerable increase in the firing rate and burst activity of the AHA neurons, which suggests that this region is sensitive to hormonal responses to stress [29].

Neuroanatomical and functional studies on the AHA have demonstrated the importance of this area in stress response and feeding and have indicated that the AHA has integrative and relay functions in the stress and feeding brain circuits. However, the changes in neuronal activity in the AHA neurons during stress and feeding in freely moving rats remain unclear. The present study was designed to investigate the firing rate and bursting activity of the cAHA neurons during consumption of palatable sucrose in non-stressful conditions and after acute stress in freely moving rats.

## Materials and Methods

### Animals

Male Sprague-Dawley rats (n = 10) were purchased from the Canadian Breeding Laboratories (St-Constant, QC, Canada). All the rats were housed in individual plastic home cages lined with wood shavings and maintained on a 12:12 dark-light cycle (with the lights on from 06:00 to 18:00), with an ambient temperature of 22 ± 1°C, and free access to tap water and the standard laboratory rat diet (Rat/Mouse/Hamster chow, 1000 Formula, 12.9 kJ/g; Agway Prolab, Syracuse, NY), unless otherwise specified. All rats were cared for and handled according to the *Canadian Guide for the Care and Use of Laboratory Animals*, and the present protocol was approved by our institutional animal care committee (Comité de protection des animaux de l'Université Laval).

### Surgery

**Fig 1:**
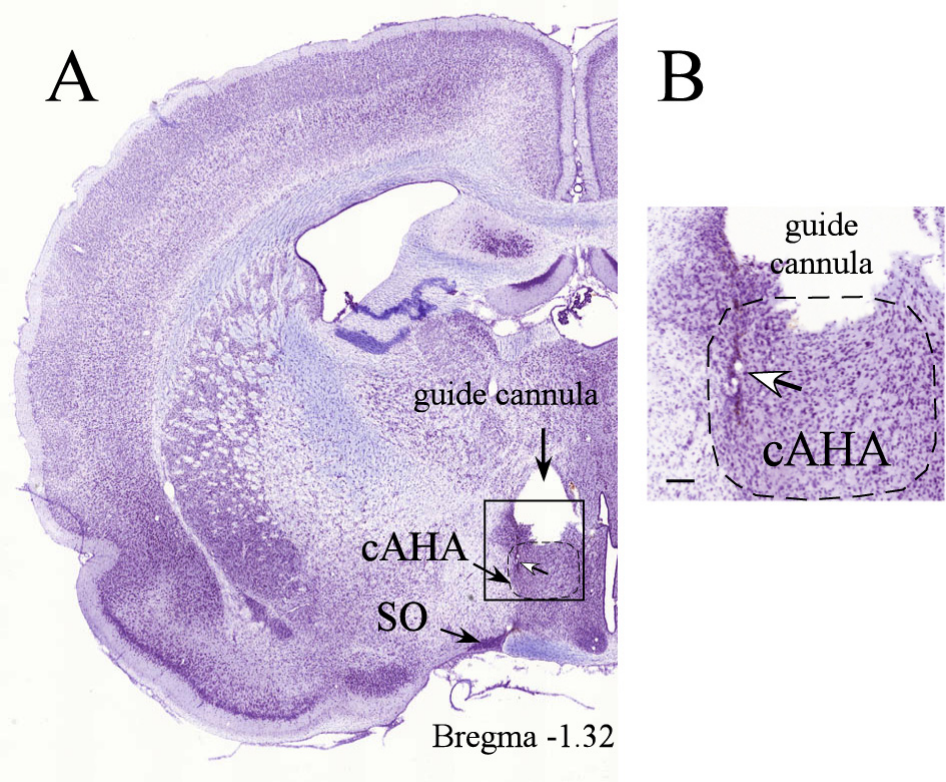
*A*, Thionine-stained coronal brain section at the level of the central nucleus of the anterior hypothalamic area (cAHA; 1.32 mm caudal to the bregma) depicting electric micro-lesions (indicated by the white arrow) at the tip of the recording electrode that was lowered 1 mm ventral from the distal edge of the guide cannula. *B*, Panel *B* is an enlargement of a square shown in panel *A*. SO, supraoptic hypothalamic nucleus. Scale bar, 200 μm.

Rats were anesthetized using 4% isoflurane mixed with 2% oxygen; the dose was gradually reduced to 2% isoflurane– 1% oxygen during the surgery. Under anesthesia, the head was shaved, eye lubricant was applied, the analgesic buprenorphine (0.02 mg/kg) was administered subcutaneously under the abdominal skin, and a mixture of lidocaine (7 mg/kg) and bupivacaine (3.5 mg/kg) was injected subcutaneously over the skull incision site and area in contact with the stereotaxic ear bars. A chronically implanted unilateral 25-gauge guide cannula was aimed at the dorsal border of the cAHA (1.32 mm caudal to the bregma, 0.6 mm lateral to the midline, and 8.6 mm ventral to the scalp) [30]. Bundles of 16 microwire electrodes (platinum-iridium, formvar coated, 25-μm diameter, with an impedance of 250–500 kΩ measured at 1 kHz; California Fine Wire, Grover Beach, CA), along with one 50-μm diameter reference electrode made of the same material and insulation (California Fine Wire, Grover Beach, CA) were lowered into the guide cannula and protruded 1 mm below the distal end of the guide cannula (Fig 1). A stainless steel wire, as the conventional ground, was shouldered on one of the supporting screws on the skull. Rats were hydrated with saline and subcutaneously administered with the anti-inflammatory analgesic meloxicam (1 mg/kg) after completion of the surgery and before they were returned to the home cages for 7 days of recovery.

### Recording of sucrose-licking events and neuronal activity in the cAHA

After 7 days of post-operative care and 2 days of habituation to an operant chamber equipped with two photo-beam lickometers (Med Asscociates Inc, VT, USA) supplied only with water, the rats were given 1 h of daily access to 10% sucrose, in addition to water, in the operant chamber in the following 2 days, in order to counter neophobia to the taste of sucrose. After the following 2 days on *ad libitum* access to chow in their home cages, rats were given 1 h of access to 10% sucrose and water in the operant chamber, during which time simultaneous acquisition of licking activity and extra-cellular multi-unit recordings in the cAHA was performed using a custom-made interface between the operant chamber and TDT multichannel acquisition system (Tucker Davis Technologies, FL, USA). This recording session provided data on sucrose licking and multi-unit activity in control, non-stressful conditions. After recordings in the non-stressful conditions, the rats were given two days of *ad libitum* access to chow in their home cages. Thereafter, the rats were subjected to mild foot shock stress (0.6 mA, 3 s duration, 4 times with an inter-shock interval of 15 s). Immediately after the stress session, the rats were placed in the operant cage, where multi-unit and licking activity were recorded during the 1-h access to 10% sucrose and water. Bottles of sucrose and water were weighed before and after the 1-h access in the operant chamber to evaluate intake. For each rat one control recording session and one recording session after exposure to stress were performed.

On completion of the experiment, the rats were deeply anesthetized (60 mg/kg ketamine and 7.5 mg/kg xylazine), and the locations of the recording electrodes were marked by passing an anodal direct current of 90 μA for 15 s through selected electrode pairs in each bundle. The rats were then euthanized under anesthesia (60 mg/kg ketamine and 7.5 mg/kg xylazine) by intracardial perfusion with saline followed by phosphate buffered 4% paraformaldehyde. The brains were removed and stored at 4°C in a phosphate buffered 4% paraformaldehyde solution before they were cut into 40-μm coronal brain sections and stained with thionin. Rats with correct electrode placement to the cAHA (n = 8) were used for statistical analysis.

### Analysis of licking activity

Lick timestamps were used to perform lick microstructure analysis. The total number of licks was determined in each 1-h recording session. Because water intake was very low (from 0 ml to 1 ml) during the 1-h assess to sucrose, the licking microstructure was analyzed only for sucrose. Sucrose-licking clusters were defined as high-frequency (6–9 Hz) licks occurring in a run of three or more licks interrupted by pauses of inter-cluster intervals (ICIs) of 3 s or longer [31]. The total number of licks, cluster number, cluster duration (s) and number of licks per cluster were calculated for each 1-h session using a custom written MATLAB script (R2010a, The MathWorks^TM^). The total meal duration (s) was calculated as the sum of all cluster durations during the 1-h access to sucrose. Lick frequency within a cluster was estimated by dividing the number of licks per cluster by cluster duration. The mean inter-lick interval (ILI, ms) was calculated by creating an inter-lick interval histogram of the total lick events during the 1-h recording session with a bin size of 1 s.

### Analysis of neuronal firing

Multi-unit activity was recorded using a 25-kHz sampling rate and signal filtering between 300 and 8000 Hz. The units with a signal-to-noise ratio of 3:1 were discriminated using a digital window discriminator. The principal component analysis spike sorter (Tucker Davis Technologies, FL, USA) was used for online sorting of captured spikes and their storage for offline analysis. Open sorter (Tucker Davis Technologies, FL, USA) was used to further refine the recorded multi-unit activity into individual, well-defined single-unit clusters using k-mean clustering. The mean waveform shape of each unit was identified using *NeuroExplorer* (Nex Technologies, Dallas, TX, USA).

**Fig 2:**
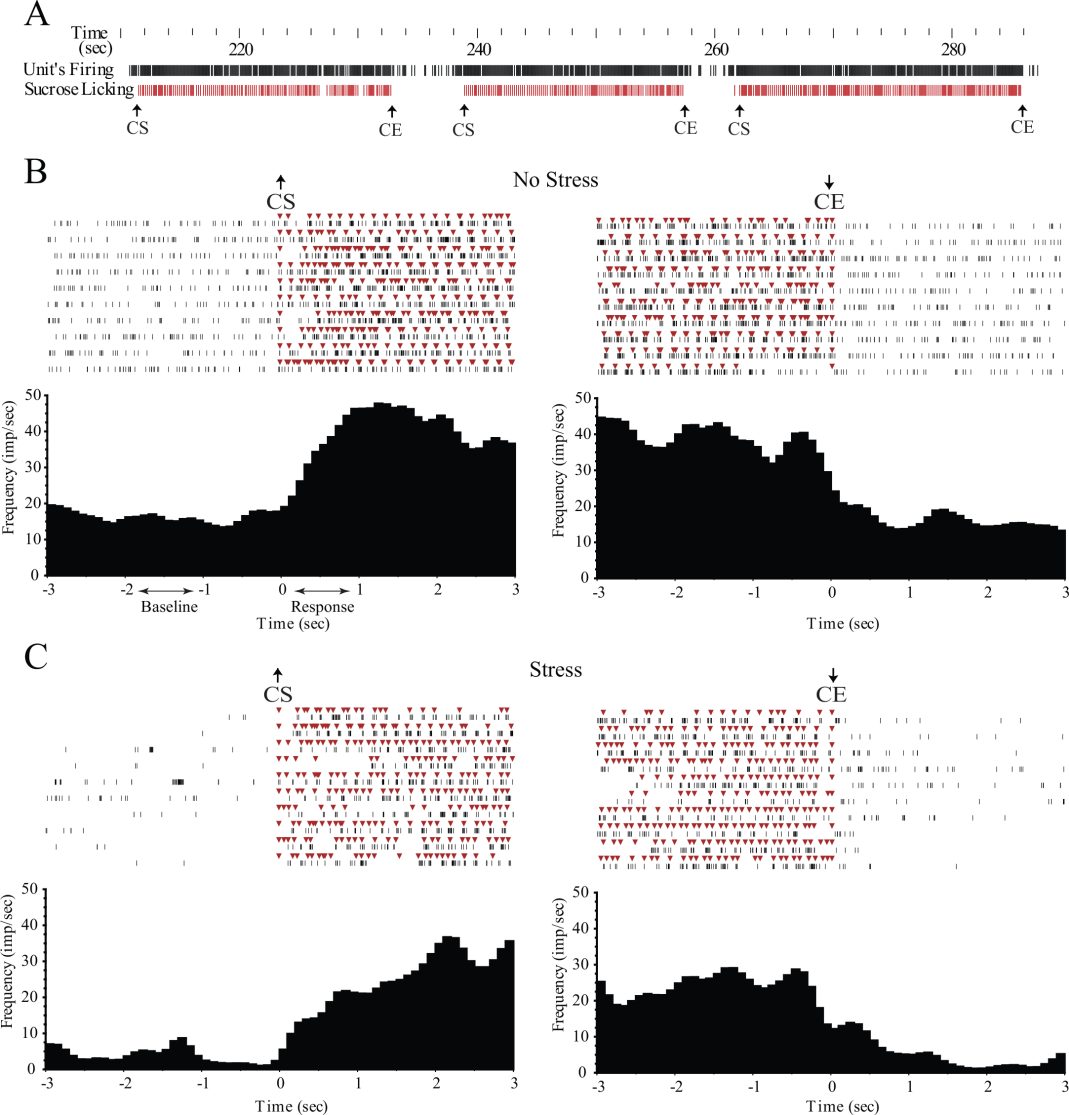
*A*, Example of simultaneous recording of sucrose-licking events and the neuronal firing of a neuron with an increased firing rate at the cluster start (CS), termed CS-excited neuron. Clustering of the sucrose-licking events was based on inter-cluster intervals (ICIs) ≥ 3 s. *B*, Peri-event histograms (PEHs) and rasters of a CS-excited neuron in non-stressful conditions. *C*, PEHs and rasters of a CS-excited neuron after exposure to stress. Note that the firing rate of the CS-excited neurons decreased at the end of the sucrose-licking clusters (CE). In the rasters, the red triangles indicate sucrose licks and the black vertical dashes indicate unit discharges. The bin duration in the PEHs was 100 ms. Bin frequency values (impulses per second) from –2 s to –1 s were used to calculate the mean baseline frequency. Bin frequency values from 0 s to +1 s were used to characterize response to the CS.

**Fig 3:**
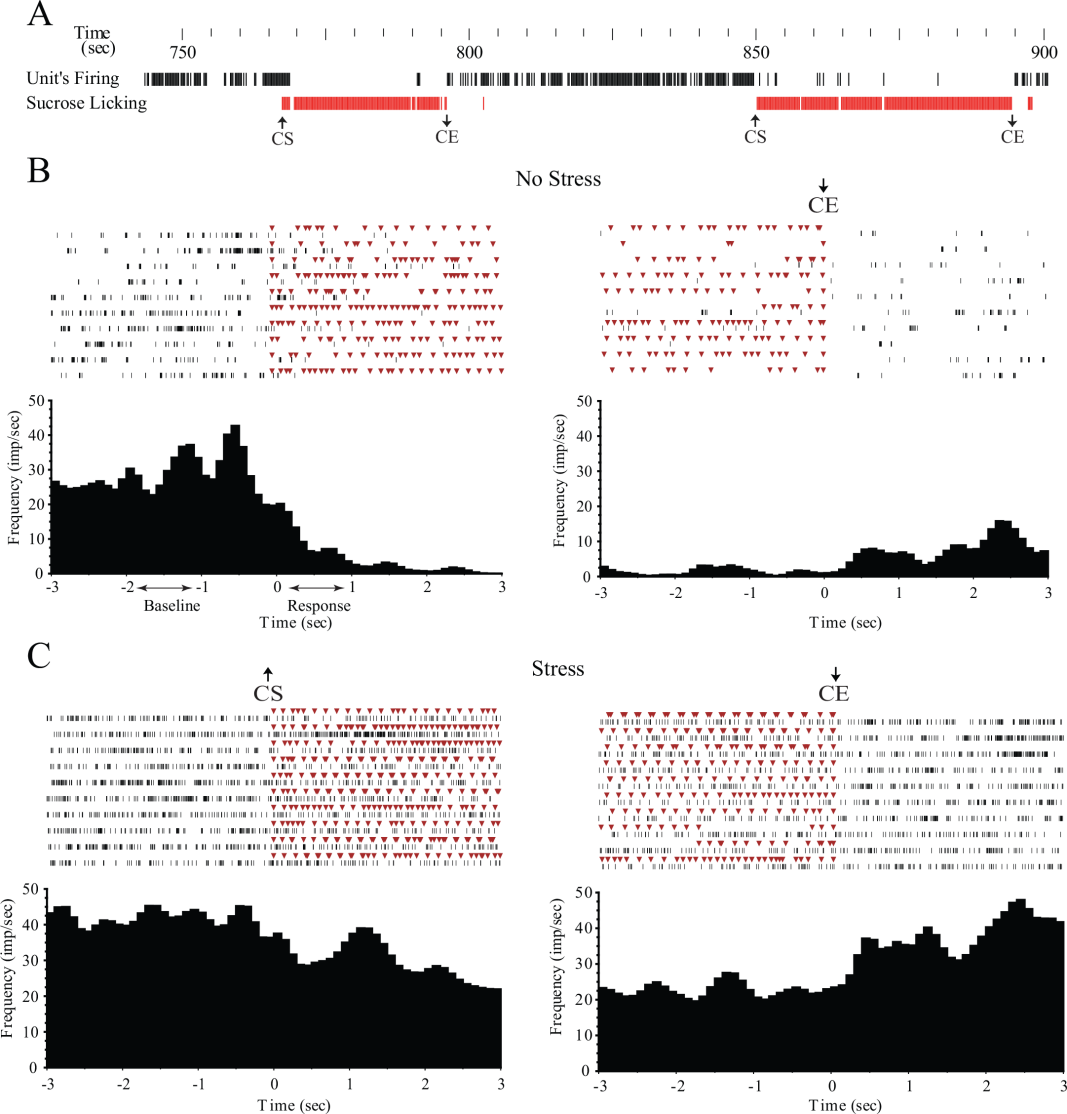
*A*, Example of simultaneous recording of sucrose-licking events and the neuronal firing rate of a neuron with decreased firing rate at the cluster start (CS), termed CS-inhibited neuron. Clustering of the sucrose-licking events was based on inter-cluster intervals (ICIs) ≥ 3 s. *B*, Peri-event histograms (PEHs) and rasters of a CS-inhibited neuron in non-stressful conditions. *C*, PEHs and rasters of a CS-inhibited neuron after exposure to stress. Note that the firing rate of the CS-inhibited neurons increased at the end of the sucrose-licking cluster (CE). In the rasters, the red triangles indicate sucrose licks and the black vertical dashes indicate unit discharges. The bin duration in the PEHs was 100 ms. Bin frequency values (impulses per second) from –2 s to –1 s were used to calculate the mean baseline frequency. Bin frequency values from 0 s to +1 s were used to characterize response to the CS.

To characterize the neurons according to sucrose licking activity, 6-s (±3 s, 100-ms bin duration) peri-event rasters and peri-event histograms (PEHs) were created by aligning the unit firing to the licking cluster start (CS) or cluster end (CE) as a reference event (Figs 2 and 3). The resulting PEHs were smoothed with a Gaussian filter (bin width = 3) to reduce fluctuation in the firing rate due to licking. The PEH bin values are given in spikes per second and represent the mean frequency across all licking clusters for each neuron.

The mean baseline firing rate (shown in Figs 2B and 3B) of each neuron was determined in the –2 s to –1 s time interval before the first lick of the cluster. The mean firing rate during the response to CS was calculated from 0 s to 1 s (Figs 2B and 3B). The baseline and response mean firing rate of all the recorded neurons was calculated for each experimental condition. Similar to the CS analysis, we also calculated statistically significant changes by using CE as a reference event. However, the majority of neurons showed similar results for the CS and CE analyses. To simplify the categories in terms of neuronal response, the final analyses were performed using only CS as the reference event, and we reported data only from this analysis.

The neurons were classified as CS-excited (excitation at CS, Fig 2) if 1 s after the CS the frequency counts (10 bin counts from 0 s to 1 s, with a 100-ms bin duration) were significantly higher (Wilcoxon signed-rank test, p< 0.05) than the frequency counts at the baseline (10 bin counts from –2 s to –1 s, with a 100-ms bin duration). The neurons were classified as CS-inhibited (inhibition at CS, Fig 3) if 1 s after the CS the frequency counts were significantly lower than the frequency counts at the baseline. If no significant difference was found between the frequencies after CS (0 s to +1 s) and at the baseline (–2 s to –1 s), the neuron was classified as CS-nonresponsive at the start of sucrose licking. A non-parametric test (Wilcoxon signed-rank test) was used because the normality test (Kolmogorov–Smirnov, p< 0.05) did not show normal distribution of the frequency counts.

To estimate the general 1-h dynamics of the firing rate of CS-classified neurons, rate histograms with a bin value of 1 s were created for each neuron for the entire 1 h of recording, with the exception of the first 2 min. The first 2 min were excluded from this analysis because during the first 2 min, system calibration and establishment of the units’ thresholds across the recording channels at the beginning of each recording sessions were performed. The mean firing rate across the 1-h recording was calculated in non-stressful and stressful conditions for CS-classified groups of neurons. The correlation between the firing rate of CS-inhibited, CS-excited and CS-nonresponsive neurons in non-stressful and stressful conditions was assessed using the non-parametric Spearman correlation test.

### Burst analysis

A fraction of the AHA neurons showed burst activity in the *in vitro* recordings and in the *in vivo* recordings under anesthesia [32,33]. To the best of our knowledge, this study is the first to present the burst analysis of the AHA neurons in freely moving rats. To examine the burst activity of the cAHA neurons, we used the Poisson surprise method [34]. In the Poisson surprise method as implemented in *NeuroExplorer* (Nex Technologies, AL, USA), a burst is detected as a group of three or more spikes with an inter-spike interval (ISI) less than half of the average ISI in the whole spike train (ISI in the burst < the average ISI/2). For each selected burst, the Poisson surprise value, that is, the negative logarithm of the probability of the occurrence of a burst in a random (Poisson) spike train [34], was calculated. The following algorithm attempted to maximize the surprise value for the burst by adding the next intervals in the spike train and removing intervals from the beginning of the burst. We used a minimum burst surprise value of 10, with which a high level of statistical significance (P < 0.00005) can be ensured for burst detection in a spike train. The Poisson surprise method is not sensitive to fluctuations in the average firing rate and inter-burst ISI [34,35]. This method was successfully used for detection of burst activity in cortical and subcortical neurons [34–39]. For each unit, several properties of bursts were determined including the burst number in the whole spike train, percentage of spikes in the bursts compared to the spikes number in the whole train, burst duration, mean spike number in the bursts and ISI of the bursts.

In addition, we investigated whether the burst start (BS) or burst end (BE) time of the cAHA neurons influenced the sucrose licking activity. We created 1-s PEHs (± 0.5 s, 100-ms bin duration) by aligning the licking events to BS or BE as a reference event. The Wilcoxon signed-rank test was used to find significant (p < 0.05) differences between the lick frequency before (5 bin counts from –0.5 s to 0 s, with a 100-ms bin duration) and after (5 bin counts from –0 s to 0.5 s, with a 100-ms bin duration) the reference event. To assess whether BS occurred simultaneously with CS, we created 500-ms (± 250 ms with a bin size of 1 ms) cross-correlograms between the BS and CS events. The higher level of the confidence interval was set to 95%.

### Statistical analysis

All results are presented as mean ± SEM. The paired Student’s *t*-test (p < 0.05) was used to compare sucrose intake and variables of the sucrose licking microstructure in non-stressful and stressful conditions. To assess whether the distribution of neuronal firing rates, responsiveness to sucrose and burst parameter values were normal, the Kolmogorov-Smirnoff test (p < 0.05) was used. The non-parametric Wilcoxon signed-rank test was used to compare the baseline and response firing rate of neurons classified according to sucrose licking clusters, and to calculate the differences in lick frequency at the firing burst start and end. The firing rate around the CS (baseline and response) of the CS-inhibited, CS-exited, and CS-nonresponsive neurons was analyzed using 2-way repeated-measures ANOVA to detect the main and interactive effects of stress and response to CS. Differences between individual groups were assessed using the Bonferroni *post-hoc* test. The 1-h mean firing rate of the CS-classified neurons was compared using the Kruskal-Wallis test following by Dunn’s multiple comparisons test. The non-parametric Spearman correlation test was used to determine the correlation between pairs of CS-classified groups. A Spearman’s rank correlation coefficient (*r*) value of >0.5 was considered to indicate a significant correlation between the pairs. The bursts in non-stressful and stressful conditions were assessed using the non-parametric Mann-Whitney test. The Pearson chi-square test was used to compare the number of neurons classified according to cluster-related activity. Results were considered significant at P values <0.05. The statistical tests were performed using *GraphPad* (GraphPad Software Inc., La Jolla CA, USA), MATLAB (R2010a, The MathWorks^TM,^ Natick, MA) and *NeuroExplorer* (Nex Technologies, AL, USA).

## Results

### Sucrose intake and lick microstructure

Sucrose intake was measured during the 1-h access to 10% sucrose in the operant chamber in the non-stressful control condition and immediately after foot shock stress. In non-stressful conditions, the rats consumed 18.6 ± 1.7 ml sucrose during the 1-h access, whereas foot shock stress significantly (p = 0.0474, paired Student’s *t*-test) decreased sucrose intake to 12.9 ± 1.7 ml (Table 1).

**Table 1:** Sucrose intake and licking microstructure during the 1-h access in rats in non-stressful conditions and after acute foot shock stress.

	No Stress	Stress
Sucrose intake (ml)	18.6 ± 1.7	12.9 ± 1.7*
Total lick number	5348 ± 778	3833 ± 487*
Total meal duration (s)	665.4 ± 85.2	563.1 ± 35.6
Mean inter-lick interval (ILI) (ms)	356.2 ± 38.1	603.5 ± 80.4*
Cluster number	19.5 ± 3.4	23.6 ± 3.4
Cluster duration (s)	41.5 ± 8.9	30.0 ± 6.5
Lick number per cluster	352.8 ± 107.3	195.2 ± 36.8
Lick frequency within cluster (Hz)	8.0 ± 0.7	6.6 ± 0.7*

Clusters were defined using inter-cluster intervals (ICIs) ≥ 3 s.

*Significantly different (paired Student’s *t*-test) compared to No Stress.

The total number of licks in the 1-h access period significantly decreased (p = 0.0339, paired Student’s *t*-test) after exposure to stress, from 5348 ± 778 licks in the non-stressful condition to 3833 ± 487 licks after stress. Stress was not found to have a significant effect on the cluster number (p = 0.2959), cluster duration (p = 0.2813), meal duration (p = 0.3512) or number of licks per cluster (p = 0.1309). Conversely, stress resulted in a significant increase (p = 0.0083, paired Student’s *t*-test) in the mean ILI and a significant decrease (p = 0.0391, paired Student’s *t*-test) in the lick frequency within clusters.

### Neuronal firing activity in relation to sucrose licking clusters

In total, 417 single neuronal units were isolated and analyzed. Among these, 231 neurons were examined in control non-stressful conditions, and 186 neurons were examined after foot shock stress. For each neuron, PEHs were created using ±3 s time windows around CS or CE (as shown in Figs 2 and 3). The clustering of licking events used for analyses of neuronal activity was based on ICI values ≥3 s, which was identical to the analyses of licking microstructure (shown in Table 1). Generally, the neurons that demonstrated an increase in firing rate in response to CS decreased their firing rate at CE (as shown in Fig 2). Conversely, the neurons that had a decreased firing rate at CS showed an increase in their firing rate at CE (Fig 3). Therefore, the analysis was based on the neuronal response at CS. The mean firing rate was calculated at the baseline (from –2 s to –1 s before CS) and during the response to CS (from 0 s to 1 s after CS) (as shown in Figs 2B and 3B).

Compared to the baseline firing rate, some cAHA neurons showed a significant (Wilcoxon signed-rank test, p<0.05) increase (CS-excited, as shown in Fig 2), decrease (CS-inhibited, as shown in Fig 3), or no change (CS-nonresponsive) in their firing rate in response to CS. In non-stressful conditions, the percentage of CS-inhibited and CS-nonresponsive neurons was relatively high (42% and 37.7%, respectively) compared to the percentage of CS-excited neurons (20.3%) (Table 2). Foot shock stress resulted in a slight decrease in the percentage of CS-inhibited neurons (to 34.4%) and an increase in the percentage of CS-excited (to 26.3%) and CS-nonresponsive neurons (to 39.2%). However, the overall effects of stress on the proportion of CS-excited, CS-inhibited and CS-nonresponsive neurons were not statistically significant (χ^2^ = 3.212, p = 0.2007, Table 2).

**Table 2:** Number (n) and percentage (%) of cAHA neurons classified according to the response to the sucrose licking cluster start (CS).

	No stress		Stress	
	n	%	n	%
CS-excited	47	20.3	49	26.3
CS-inhibited	97	42	64	34.4
CS-nonresponsive	87	37.7	73	39.2

### Effects of stress on the firing rate of CS-classified neurons

**Fig 4:**
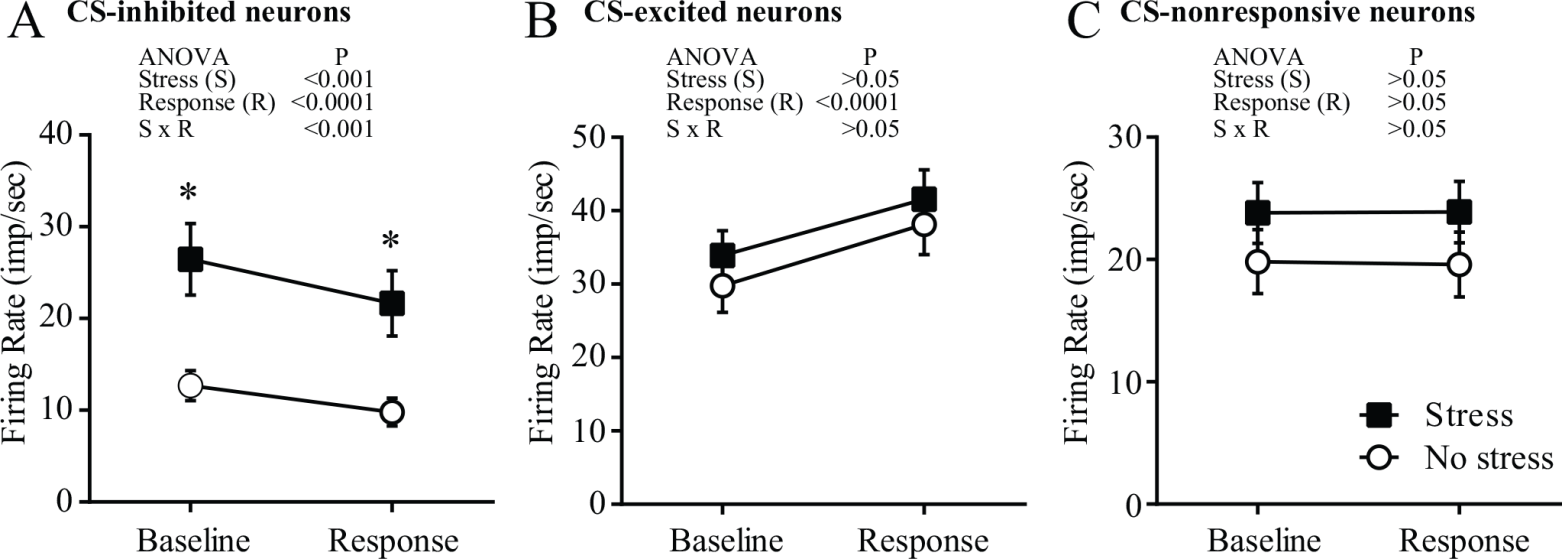
The firing rate of cAHA neurons that significantly decreased (CS-inhibited, *A*) or increased (CS-excited, *B*) their firing rate in response to the licking cluster start (CS) or were non-responsive to CS (CS-nonresponsive, *C*) in non-stressful (white circles) or stressful (black squares) conditions. The baseline firing rate was measured from –2 s to –1 s before CS, and the response to CS was calculated from 0 s to 1 s after CS. Two-way ANOVA was used to analyze the main and interactive effects of stress (stressful *vs* non-stressful conditions) and the response to CS (baseline *vs* response time windows). *Significantly (p < 0.05, Bonferroni multiple comparison test) different from the non-stressful condition within the same time window.

Two-way ANOVA revealed a significant effect of stress (F_1,159_ = 12.75, p < 0.001), response to CS (F_1,159_ = 216.3, p < 0.0001) and their interaction (F_1,159_ = 13.47, p < 0.001) on the neuronal firing rate of CS-inhibited neurons (Fig 4A). A *post-hoc* test demonstrated that stress resulted in a significant increase in the firing rate of these neurons during the baseline (p < 0.001) and response (p < 0.01) time windows, in comparison to the firing rate in non-stressful conditions (Fig 4A).

Conversely, two-way ANOVA did not show any significant effect of stress (F_1,94_ = 0.5105, p = 0.4767) on the firing rate of CS-excited neurons (Fig 4B). Similarly, stress was not observed to affect (F_1,158_ = 1.263, p = 0.2628) the firing rate of CS-nonresponsive neurons (Fig 4C). Neither CS (F_1,158_ = 0.7874, p = 0.3762) nor the interactive effect of CS and stress (F_1,158_ = 3.147, p = 0.078) had the significant effects on the firing rate of CS-nonresponsive neurons, according to two-way ANOVA.

**Fig 5:**
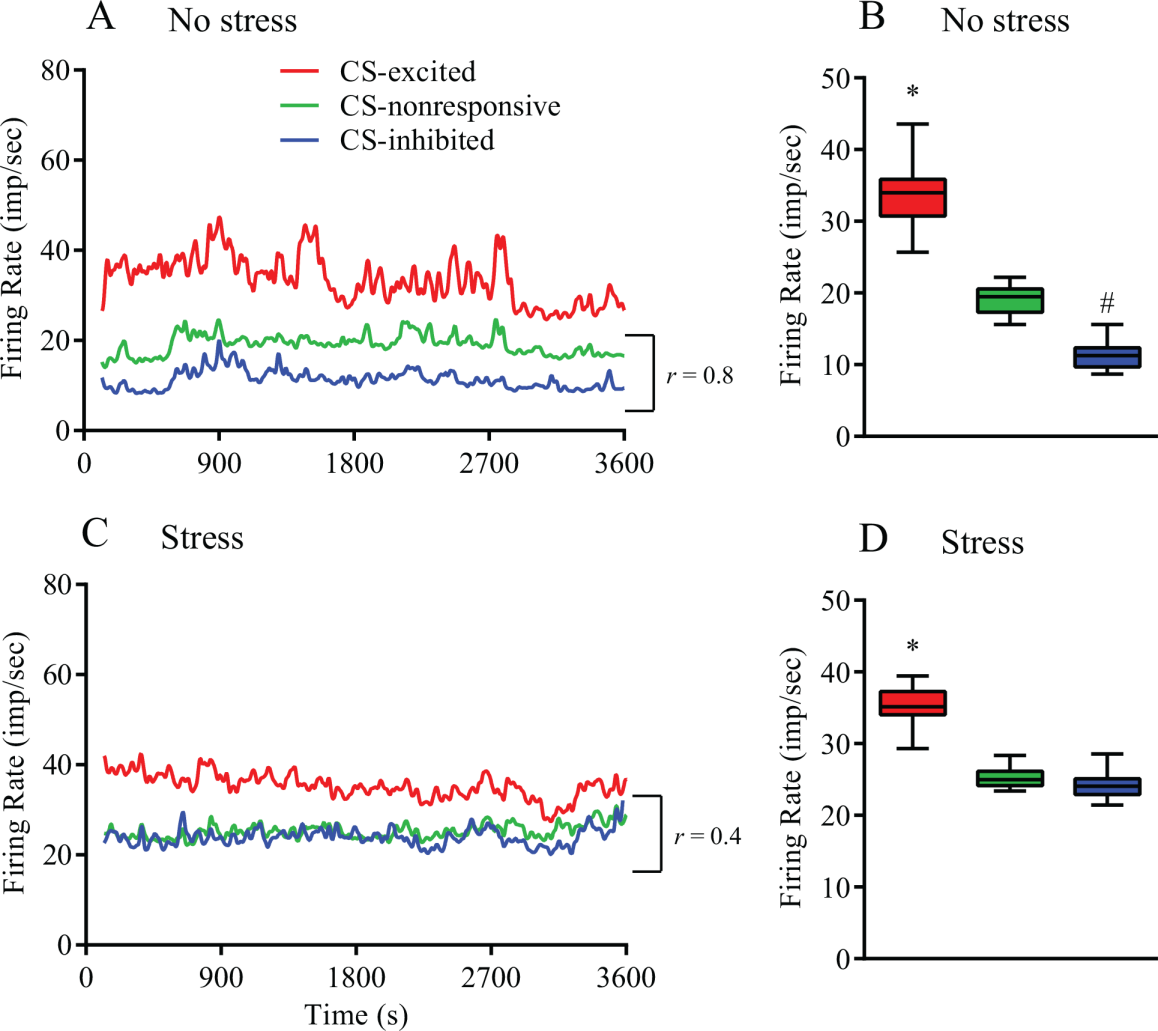
One-hour dynamics of the firing rate (bin value in the line graphs, 1 s) of the cAHA neurons that significantly increased (CS-excited, red) or decreased (CS-inhibited, blue) their firing rate in response to the licking cluster start (CS) or were non-responsive to the CS (CS-nonresponsive, green) in non-stressful conditions (*A*) or immediately after foot shock stress (*C*). Spearman’s rank correlation coefficient (*r*) shows a strong correlation between the firing rate of CS-inhibited and that of CS-nonresponsive neurons in non-stressful conditions (r > 0.05, *A*) but such a correlation was not observed after stress (r < 0.05, *C*). The 1-h mean firing rate of the CS-excited (red), CS-nonresponsive (green), and CS-inhibited (blue) neurons was compared using the non-parametric Kruskal-Wallis test followed by a *post hoc* test in non-stressful conditions (B) and after foot shock stress (D). *Significantly (p < 0.05; Dunn’s multiple comparisons test) higher compared to all the other groups. ^#^Significantly lower compared to all the other groups.

Additional analyses were performed to determine whether stress-induced increase in the firing rate of CS-inhibited neurons was not limited to the CS-related time windows. To estimate the overall dynamics of the firing rate of CS-classified neurons, rate histograms were created for each neuron for the entire 1-h recording period with a bin value of 1 s (Fig 5A and 5C). The mean firing rate across the 1-h recording period was calculated in non-stressful (Fig 5B) and stressful (Fig 5D) conditions for CS-classified neuronal groups.

In non-stressful conditions, the mean firing rate of the CS-excited neurons estimated during the 1-h recording was significantly higher than the mean firing rate of the CS-nonresponsive (p < 0.0001) and CS-inhibited (p < 0.0001) neurons (Fig 5B). In addition, the firing rate of the CS-inhibited neurons was significantly (p < 0.0001) lower than that of the CS-nonresponsive neurons (Fig 5B). Interestingly, in non-stressful conditions, a significant correlation was observed between the firing rate of the CS-inhibited and that of the CS-nonresponsive neurons (r = 0.787, Spearman correlation test), but this correlation was not observed between the CS-nonresponsive and CS-excited neurons (r = 0.478), or between the CS-inhibited and CS-excited neurons (r = 0.463) over the 1-h recording period (Fig 5A).

Foot shock stress resulted in an increase in the firing rate of the CS-inhibited neurons to levels comparable to those in CS-nonresponsive neurons (p = 0.1201, Fig 5D) and disrupted the correlation between the firing rate of CS-inhibited and CS-nonresponsive neurons (r = 0.459, Fig 5C). Similarly, other neuronal groups did not demonstrate a correlation between the firing rate under stress (r = 0.098, CS-excited *vs*. CS-nonresponsive; r = 0.278, CS-inhibited *vs*. CS-excited). The *p* values of Spearman correlation tests for all pairs of neuronal groups in non-stressful and stressful conditions was <0.0001; this shows that the results of the correlation tests were not due to random sampling. Similar to the non-stressful condition, after stress the CS-excited neurons showed a higher firing rate compared to other neuronal groups (p < 0.0001, CS-excited *vs*. CS-nonresponsive; p < 0.0001, CS-excited *vs*. CS-inhibited; Fig 5D).

### Burst firing of cAHA neurons

**Fig 6:**
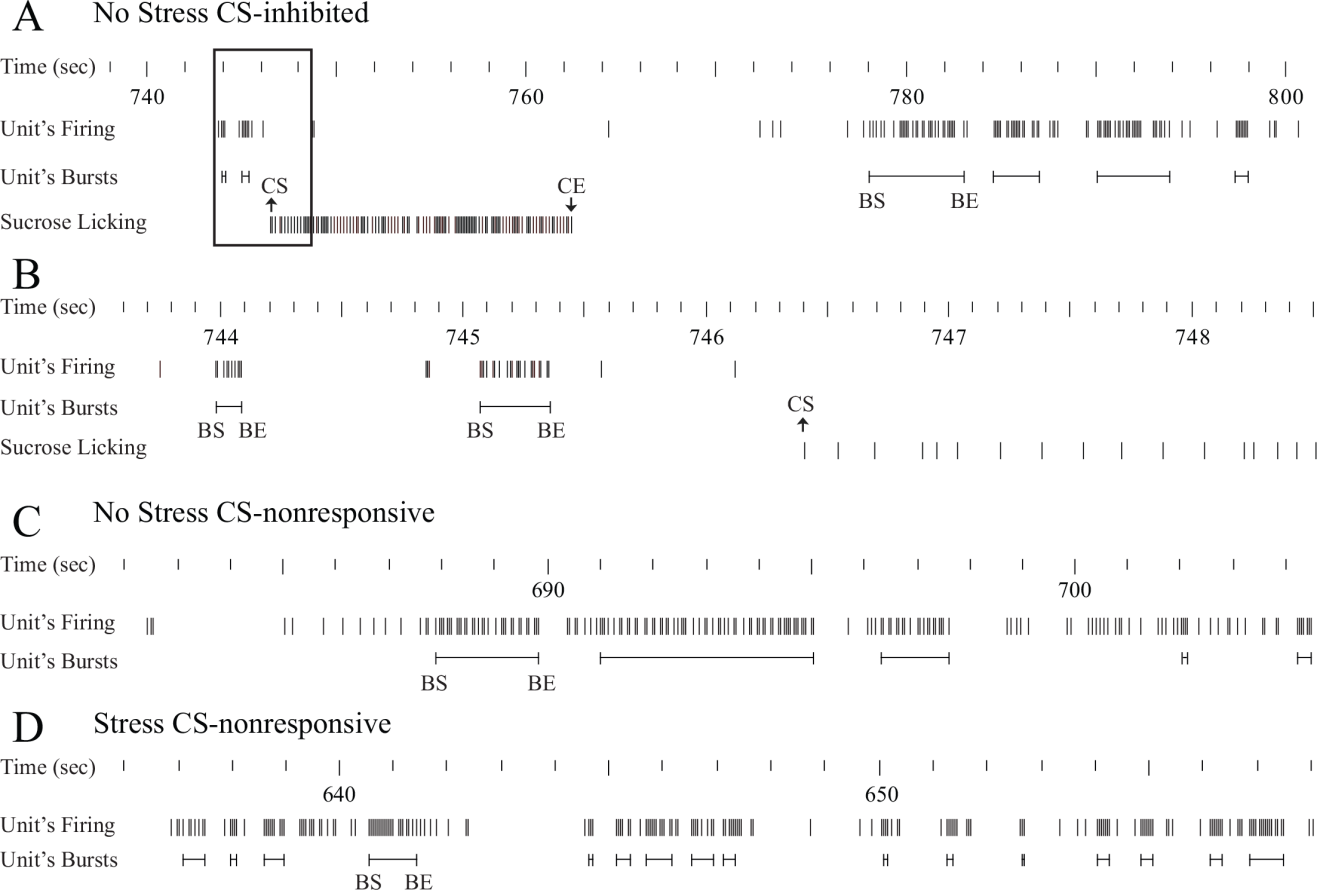
*A*, An example of burst firing of a CS-inhibited neuron that decreased its firing rate at the cluster start (CS) of sucrose licking. *B*, Enlargement of a time window depicted by a quadrant in panel *A*. *C*, Burst firing of a CS-nonresponsive neuron in non-stressful conditions. *D*, Burst firing of a CS-nonresponsive neuron after foot shock stress. The bursts are depicted by horizontal lines, with some burst starts (BS) and burst ends (BE) indicated on the line. CE, cluster end of sucrose licking.

The Poisson surprise method was used to detect individual bursts in the spike trains of recorded cAHA neurons (Fig 6).

There was no significant effect of the BS or BE time on sucrose licking events for any of the neurons recorded during non-stressful and stressful conditions, as assessed by the Wilcoxon signed-rank test (α = 0.05) in PEH (aligned to BS or BE) of licking events. In addition, cross-correlograms between BS and CS events for each neuron in non-stressful and stressful conditions did not show significant correlation between these events. Therefore, in the burst firing analyses, we took into consideration the entire 1-h recording of the spike train rather than responses around the lick clusters to assess the differences in burst parameters during non-stressful and stressful conditions. A small fraction of CS-inhibited (1 out of 97 neurons or 1.1% neurons in the non-stressful condition; 1 out of 64 neurons or 1.6% neurons in the stressful condition), CS-excited (2 out of 47 neurons or 4.3% neurons in the non-stressful condition; 2 out of 49 neurons or 4.1% neurons in the stressful condition), and CS-nonresponsive (0 out of 87 neurons or 0% neurons in the non-stressful condition; 2 out of 73 neurons or 2.7% neurons in the stressful condition) neurons failed to show any burst-firing episode during the entire 1-h recording and were therefore removed from the burst analysis.

In addition to the burst number, we calculated the percentage of spikes in bursts during the 1-h recording, burst duration, mean spikes in the bursts and ISIs in bursts (Table 3).

**Table 3:** Burst parameters of the cAHA neurons classified according to the responses to cluster start (CS) of sucrose licking during 1-h access to sucrose in non-stressful conditions or after foot shock stress.

Burst Parameters	CS-Inhibited		CS-Excited		CS-Nonresponsive	
	No stress	Stress	No stress	Stress	No stress	Stress
Burst number	118.1 ± 14.8	111.0 ± 16.3	119.3 ± 27.1	38.3 ± 6.7*	71.8 ± 7.7	77.8 ± 17.5*
% Spikes in burst	40.1 ± 2.8	32.9 ± 3.6	18.4 ± 3.7	9.9 ± 3.3	27.4 ± 2.8	12.3 ± 2.5*
Burst duration (s)	1.6 ± 0.2	1.2 ± 0.2	0.6 ± 0.1	0.8 ± 0.2	2.1 ± 0.8	0.8 ± 0.2*
Mean spikes in burst	48 ± 5.3	75.9 ± 11.1	44.4 ± 5.8	43.8 ± 4.4	61.0 ± 8.0	34.2 ± 1.2*
ISI in burst (ms)	45.9 ± 7.7	32.3 ± 5.5*	14.7 ± 3.3	21.1 ± 5.4	54.4 ± 9.1	26.9 ± 5.2*

*Significantly (p < 0.05) different compared to No Stress within the same group of neurons.

The CS-inhibited neurons failed to exhibit any significant changes in burst number (p = 0.3348) or other parameters (percentage of spikes in bursts, p = 0.2231; burst duration, p = 0.0951; mean spikes in the burst, p = 0.0785); however, a significant decrease (p < 0.05) was observed in the ISIs within bursts following stress (Table 3). As the number of bursts and mean spikes per burst were comparable, it seems that stress elevated the firing rate of CS-inhibited neurons by decreasing ISIs within the bursts.

Conversely, in the CS-excited neurons, the burst number significantly (p < 0.001) decreased following stress (Table 3). The percentage of spikes in the burst (p = 0.0654), burst duration (p = 0.8539), mean number of spikes in the bursts (p = 0.9172) and ISIs in the bursts (p = 0.9665) remained statistically comparable between the non-stressful and stressful conditions.

CS-nonresponsive neurons exhibited robust changes in all the burst parameters following stress. In fact, stress significantly (p < 0.05) increased the number of burst but decreased the percentage of spikes in the bursts (p < 0.0001), burst duration (p < 0.0001), mean number of spikes in the bursts (p < 0.001), and ISIs within bursts (p = 0.0188) in the CS-nonresponsive neurons (Table 3).

## Discussion

We investigated real-time neuronal activity in the cAHA in freely moving rats during sucrose intake in non-stressful conditions and after foot shock stress. Acute stress resulted in a significant decrease in the consumption of sucrose, along with a decrease in the total number of licks and lick frequency within sucrose licking clusters. We demonstrated for the first time that the cAHA contains sucrose-responsive CS-excited and CS-inhibited neurons as well as CS-nonresponsive neurons. Stress affected the firing characteristics of all the neuronal groups by increasing the firing rate of the CS-inhibited neurons and by changing the burst activity of all types of neurons. In addition, stress disrupted the correlation between the firing rate of CS-inhibited and CS-nonresponsive neurons that was observed in non-stressful conditions. These results suggest that the cAHA can integrate signals related to stress and the intake of palatable food.

Acute stress activates the HPA axis [40,41] and leads to short- and long-term neuronal and physiological adaptations [42,43] including alterations in food intake [44,45]. In the present study, acute foot shock stress caused a significant decrease in sucrose intake during the 1-h sucrose access sessions immediately following foot shock. This result is in accordance with the strong anorectic effects of acute stress demonstrated in rats [45–48]. In particular, sucrose intake was significantly decreased after an acute session of foot shock stress [45]. Similar to a previous report [45], the present study did not detect any effects of acute foot shock stress on the number and duration of sucrose licking clusters. However, a decrease in the volume of sucrose consumed was induced by foot shock stress and was accompanied by a significant decrease in the total number of licks and longer ILIs. Accordingly, lick frequency within sucrose-licking clusters was significantly decreased by stress. A decrease in the total number of licks and lick frequency within the licking clusters may reflect a decrease in the hedonic value of sucrose induced by acute stress [31,49–53].

A real-time multi-unit extracellular recording in the cAHA revealed three distinct subpopulations of neurons that increased (CS-excited), decreased (CS-inhibited) or did not change (CS-nonresponsive) their firing rate at the start of the sucrose licking clusters. The majority of the examined cAHA neurons were either CS-inhibited (42%) or CS-nonresponsive (37.7%); only ~20% of the neurons exhibited CS-excited characteristics. Therefore, this region seems to be populated predominantly by CS-inhibited and CS-nonresponsive neurons. The relatively high amount of CS-inhibited neurons compared to CS-excited neurons may reflect the important role of AHA inhibition in feeding. In fact, direct inhibition of AHA by the GABA_A_ receptor agonist muscimol elicited feeding in satiated sheep [18]. Interestingly, acute stress specifically increased the firing rate of the CS-inhibited neurons, which suggests that CS-inhibited neurons in the cAHA are an important target of stress, and increased firing rate of these neurons may be involved in the anorectic effects of stress. Stress may activate the AHA neurons through local hypothalamic inputs and via the direct efferents from the limbic regions sensitive to stress, including glutamatergic neurons of the subiculum [1,3–5]. In addition, the AHA is sensitive to the peripheral stress hormone corticosterone, which increases the firing rate and burst activity of AHA neurons [29]. A stress-induced increase in the expression of GAD65 in the AHA may also reflect neuronal activation in this brain region triggered by stress [28]. Interestingly, access to highly palatable food significantly decreased stress-induced activation of GAD65 expression in the AHA [28]; this is in agreement with the present results that intake of palatable food may produce inhibitory effects in a large population of AHA neurons. GAD65 is a rate-limiting enzyme responsible for GABA synthesis [54]. The AHA contains a prominent group of GABAergic neurons [11] that densely project to the perifornical LHA [1]. The perifornical LHA expresses orexigenic neuropeptides, such as melanin-concentrating hormone and orexin [55], and inactivation of this area decreases food intake and reward-seeking behaviors [56–58]. It seems most likely that the CS-inhibiting neurons in the cAHA are GABAergic neurons. If the CS-inhibiting neurons project to the perifornical LHA, inhibition of these neurons at the start of sucrose-licking clusters would disinhibit the LHA neurons that favor sucrose intake. A relative increase in the firing rate of these neurons during stress may inhibit the LHA neurons, which will enhance the anorectic effects of stress. In fact, it has been reported that GABA_A_ receptor blockade in the LHA elicits food intake while activation of GABA_A_ receptors in the LHA suppresses feeding behavior [59,60].

We also investigated possible co-modulation of the activity of CS-related neuronal groups in the cAHA by performing a correlation analysis of the firing rate of neurons within 1-h recordings in stressful and non-stressful conditions. CS-excited neurons displayed independent firing rate dynamics that were reflected by its poor correlation with CS-inhibited and CS-nonresponsive neurons. Conversely, CS-nonresponsive and CS-inhibited neurons showed a strong positive correlation (r = 0.8) during control non-stressful conditions. Stress disrupted the correlation between the CS-inhibited and CS-nonresponsive neurons (r = 0.4), and under stressful conditions, no significant correlation was detected between the CS-related groups in the cAHA. Neuronal groups can synchronize their activity with a precision of a few milliseconds if they are activated by a common stimulus [61,62]. Correlated activity of the cortical neuronal populations was related to motor behavior during behavioral tasks in monkey [63] and licking activity in mice [64]. Whether correlated firing of the CS-inhibited and CS-nonresponsive neurons in non-stressful conditions is related to higher sucrose intake and whether disruption of this correlation reflects stress-induced hampering of sucrose-licking activity remains to be delineated.

To further characterize the cAHA neurons, we analyzed the burst activity of CS-related neurons in non-stressful conditions and after foot shock stress. Bursting is a particular firing pattern wherein a neuron generates the action potentials in brief high-frequency discharges separated by periods of silence [65]. Bursting represents a particular mode of neuronal signalling and is involved in the transmission of behaviorally relevant sensory information [66,67]. Burst activity may depend on the intrinsic properties of neurons [65]; however, in freely behaving animals, the burst activity represents integration of intrinsic properties and high-frequency sensory inputs [66]. In *in vitro* recordings, burst activity was detected in the majority of the AHA gonadotropin-releasing hormone (GnRH) neurons [32,33]. However, only 15% of the GnRH AHA neurons showed a burst pattern of firing under pentobarbital-induced anesthesia in mice [33]. In *in vitro* recordings of GABAergic AHA neurons, the proportion of burst-firing neurons was reported to increase from 13% in control conditions to 64% on activation of the mineralocorticoid receptor (MR) [29]. During wakefulness, the neurons frequently display irregular firing with bursting interspersed in tonic activity or silence [66]. Because of the general irregular mode of firing in freely moving rats, we did not attempt to classify the cAHA neurons according to their burst pattern, but we included in the analyses all bursts detected during the 1-h recording. Only a small fraction (up to 4.3%) of cAHA neurons failed to show any burst-firing episode in the entire 1-h recording. The relatively high proportion of bursting neurons may be explained by the active efferent circuitry innervating the cAHA in awake, freely moving rats. The burst characteristics of the cAHA neurons obtained in this study are in accordance with those described for the bursts in the AHA neurons [29,32]. Thus, burst occurrence, duration, and ISI in bursting AHA neurons in *in vitro* recordings were 0.82 bursts/min, 1.9 s, and 16.8 ms, respectively [29,32]. In our study, the burst frequency was 38.3–118.1 bursts/h or 0.6–1.9 bursts/min; the burst duration, 0.6 to 2.1 s; and ISIs, 14.7 to 54.4 ms. However, the number of spikes per burst and firing frequency within the bursts were about 10 times lower in *in vitro* recordings [32] than in freely behaving rats in this study. Lower firing frequency in recordings from the hypothalamic brain slices than in recordings from freely moving animals may be explained by a dramatic reduction in the frequency of postsynaptic potentials in the hypothalamic neurons in the *in vitro* recordings compared to the *in vivo* experiments [68,69]. The average number of spikes per burst found in the present study in the cAHA neurons in freely moving rats is comparable to that reported in the prefrontal cortical neurons in awake rats [35,70].

Stress significantly reduced the ISIs in bursts of the CS-inhibited neurons. This finding is in agreement with a significant increase in the firing rate of the CS-inhibited neurons induced by stress. In the CS-excited neurons, stress did not change burst parameters such as burst duration, number of spikes per burst and ISIs, but it significantly reduced the number of bursts. The burst activity of the CS-nonresponsive neurons was considerably affected by stress, as evidenced by an increase in burst number and a significant decrease in burst duration and number of spikes per burst. Stress-induced changes in burst activity in the AHA neurons may depend on changes in the intrinsic properties of these neurons and the altered balance between the excitatory and inhibitory inputs to the AHA. T-type and L-type calcium channels are important for triggering intrinsic burst firing in the AHA neurons [71,72]. Activation of the mineralocorticoid receptors enhanced the amplitude of calcium currents and the expression of the subunits of T-type and L-type calcium channels, and significantly increased the proportion of bursting AHA neurons in *in vitro* recordings [29,73]. Hence, a stress-induced increase in plasma corticosterone may affect the bursting of AHA neurons via glucocorticoid receptors. In addition, inhibitory and excitatory postsynaptic currents may shape burst activity and increase the firing variability of the hypothalamic neurons [74]. Excitatory glutamatergic input to the AHA neurons mainly descends from the subiculum, while the inhibitory GABAergic innervation of the AHA principally originates from the BNST, VMH, and LS [3].

It has been shown that disynaptic GABAergic output from the LS to the PVN via the AHA mediates anxiety induced by optogenetic stimulation of the LS [2]. Although the net inhibition or excitation of the AHA neurons by the GABAergic input depends on the prevailing chloride gradient and the balance between the GABA_A_ and GABA_B_ receptors on the AHA neurons [32,75], activation of GABAergic output from the LS to the AHA was found to inhibit the AHA neurons and increase the plasma corticosterone [2]. Accordingly, activation of the CS-excited AHA neurons during sucrose licking may inhibit activity of the hypophysiotropic PVN neurons if CS-excited AHA GABAergic neurons directly innervate the parvocellular PVN. Excitation of this inhibitory input to the PVN may contribute to the inhibitory effects of palatable food on the activity of the hypophysiotropic PVN neurons and HPA axis [47,76–79]. Conversely, a dramatic decrease in the burst activity of the CS-excited neurons during stress may result in disfacilitation of inhibition in the PVN. In fact, there is evidence that a population of the GABAergic AHA neurons directly project to the parvocellular PVN [2]. However, whether these neurons are CS-excited neurons remains to be elucidated. A general increase in burst activity was associated with facilitation of synaptic release and temporal summation of postsynaptic potentials [80,81]. Dramatic alterations in all the burst characteristics of the CS-nonresponsive neurons and their inactivity in relation to sucrose intake suggest these neurons play a specific role in mediating stress-related signals to the brain regions regulating stress response, such as the hypophysiotropic PVN, periaqueductal gray and neurons of the medullary reticular formation [1,2].

In this study we used a moderate foot shock stress that represents a well-established model of stress that triggers activation of the HPA axis [82,83] and the sympathoadrenal system [84]. According to the pattern of brain activation induced by foot shock stress, this experimental condition was considered as a model of generalized neurogenic or psychological stress induced by both physical and emotional components [85–87]. Physical components of electrical foot shock implicates initial activation of the nociceptive pathway [88]; and long-term (60-min) nociceptive stimulation strongly activates the neurons in the anterior hypothalamus [89]. In the present study we used brief shock impulses (3-s duration) of mild intensity (0.6 mA) that did not lead to cutaneous tissue damage or prolonged pain experience. Therefore, the long-term (minute to hours) effects on feeding behavior were most probably dependent on the general stress response attributed to foot shock stress [85,86]. The future experiments involving both noxious and non-noxious stressors should help to better understand the stress-specific responses of the AHA.

In summary, we found three types of neurons in the cAHA that respond differentially to voluntary sucrose intake by an increase (CS-excited), decrease (CS-inhibited) or no change (CS-nonresponsive) in their firing rate at the start of sucrose licking. Foot shock stress induced an increase in the firing rate of the CS-inhibited neurons by decreasing ISIs within the burst firing of these neurons. This increase in the stress-induced firing rate of the CS-inhibited neurons was accompanied by a disruption of the correlation between the firing rate of CS-inhibited and CS-nonresponsive neurons that was observed in non-stressful conditions. Stress did not affect the firing rate of the CS-excited and CS-nonresponsive neurons. However, stress induced a change in the pattern of burst firing of the CS-excited and CS-nonresponsive neurons by decreasing and increasing the burst number in the CS-excited and CS-nonresponsive neurons, respectively. In addition, stress significantly shortened burst duration, and decreased the number and percentage of spikes and ISIs within the bursts in the CS-nonresponsive neurons, making these neurons particularly sensitive to stress but not to sucrose intake. Therefore, the cAHA is not a passive relay structure that transmits feeding- and stress-related signals, but rather, it is a brain region that contains neurons that modulate their activity in response to stressful conditions and food intake. The sensitivity of the cAHA neurons to stress- and food-related signals suggests that these neurons are active players in the stress- and feeding-regulated circuitries.
